# Osmotic Demyelination Syndrome Associated With Severe Acute Hypernatremia Secondary to Poorly Controlled Central Diabetes Insipidus

**DOI:** 10.7759/cureus.93048

**Published:** 2025-09-23

**Authors:** Fatimazahra Haddari, Abderrahim Wakrim, Mounir Salek, Soukaina Wakrim, Hicham Nassik

**Affiliations:** 1 Anesthesia and Critical Care, Souss Massa University Hospital Center, Ibn Zohr University, Faculty of Medicine and Pharmacy, Agadir, MAR; 2 Radiology, Souss Massa University Hospital Center, Ibn Zohr University, Faculty of Medicine and Pharmacy, Agadir, MAR

**Keywords:** central diabetes insipidus, desmopressin, hypernatremia, intensive care unit, osmotic demyelination syndrome

## Abstract

Osmotic demyelination syndrome is a rare disorder caused by destruction of the myelin sheath, particularly in the pontine region, generally secondary to rapid osmotic variations. Osmotic demyelination syndrome due to hypernatremia is rarely described in the literature. The prognosis has improved thanks to a better understanding of its pathophysiology and advances in diagnosis and therapy. In this article, we report the case of centropontine and extrapontine myelinolysis associated with severe acute hypernatremia at 181 mEq/l secondary to abrupt discontinuation of desmopressin prescribed in the setting of central diabetic insipidus complicating pituitary surgery in a 58-year-old man. The evolution was favorable after reintroduction of desmopressin and correction of hypernatremia.

## Introduction

Osmotic demyelination syndrome, or centropontine and extrapontine myelinolysis, is a rare condition characterized by the occurrence of neuron-sparing myelin sheath destruction [1], particularly in the pontine region. The condition was first described by Adams et al. in 1959 in malnourished alcoholics suffering from hyponatremia [2,3]. Around 10% of cases of centropontine myelinolysis are associated with extrapontine myelinolysis. Whereas centropontine myelinolysis causes quadriparesis with encephalopathy, extrapontine myelinolysis can manifest itself with a variety of symptoms, including mutism, dysarthria, abnormal behavior, and Parkinsonism [2,4]. It is often the consequence of a rapid osmotic change, mainly an accelerated correction of hyponatremia [5]. However, its occurrence in the context of other osmotic disorders, such as hyper- and hypoglycemia and hypernatremia, is increasingly recognized [6]. In this article, we describe the case of a patient who presented with centropontine and extrapontine myelinolysis, with typical lesions on magnetic resonance imaging, in a context of severe hypernatremia at 181 mEq/l after abrupt discontinuation of desmopressin therapy prescribed in the setting of central diabetes insipidus, with a good evolution after resuscitative management and resumption of desmopressin therapy.

## Case presentation

We report the case of a 58-year-old patient with a history of surgery, two months prior to admission, for pituitary adenoma, complicated by neurogenic diabetes insipidus, treated with desmopressin with poor compliance. Following the abrupt discontinuation of his desmopressin treatment, the patient developed rapidly progressive ascending tetraparesis over four days, with acute urinary retention, indicating urinary catheterization at the time of his emergency consultation. On admission to intensive care, the neurological examination revealed a patient with a Glasgow Coma Scale (GCS) of 12/15 (eye opening (E) = 3; verbal response (V) = 3; motor response (M) = 6), dysarthric with symmetrical, reactive pupils, tetraparesis with a motor deficit rated at 3/5 in the upper limbs and 1/5 in the lower limbs, with meningeal stiffness, abolished osteotendinous reflexes in both lower limbs, a cutaneous-plantar reflex in bilateral extension, and preserved sensitivity with no associated seizures. The patient was dehydrated, with blood pressure at 148/97 mmHg, heart rate at 121 bpm, polypneic at 40 cpm, 94% saturated with room air, apyretic at 37.7°C, capillary blood glucose at 0.85 g/l, and hourly diuresis measured at 1.6 ml/kg/hr. The initial laboratory work-up (Table 1) showed a natremia of 181 mEq/l, a chloremia of 143 mEq/l, a kalemia of 3.28 mEq/l, a urea of 1.02 g/l, a creatinemia of 17.3 mg/l, a hemoglobin of 14 g/dl, a white blood cell count of 15,250 elements/mm³, a C-reactive protein (CRP) of 86 mg/l, and platelets of 163,000 elements/mm³.

**Table 1:** Clinico-biological evolution through ICU stay ICU: intensive care unit; ND: not done.

Parameters	J0 on admission to the ICU	J1	J3 introduction of desmopressin 120 µg /day	J6	J9	J10 desmopressin 90 µg /day	J14 ICU discharge	J30
Hemoglobin (g/dl)	14	12.3	12	10.8	11	11.3	11.5	12.1
White Blood Count (e/mm³)	15,250	12,520	11,700	10,240	11,700	12,000	11,500	9,400
Platelets (e/mm³)	163,000	159,000	131,000	145,000	208,000	328,000	261,000	235,000
Natremia (mEq/l)	181	169.9	160	150	145	132	140	138
Kalemia (mEq/l)	3.28	3.31	3.95	4.38	3.8	4.05	4	3.89
Chloremia (mEq/l)	143	136	123	118	107	94	99	99
Urea (g/l)	1.02	0.88	0.77	0.61	0.54	0.27	0.33	0.35
Creatinine (mg/l)	17.3	15.1	13	8.47	6.6	5.9	6.31	5.53
Natriuresis (mmol/d)	ND	38	ND	ND	80	ND	ND	ND
Urinary Osmolality (mOsmol/l)	ND	240	ND	ND	290	ND	ND	ND

**Figure 1:**
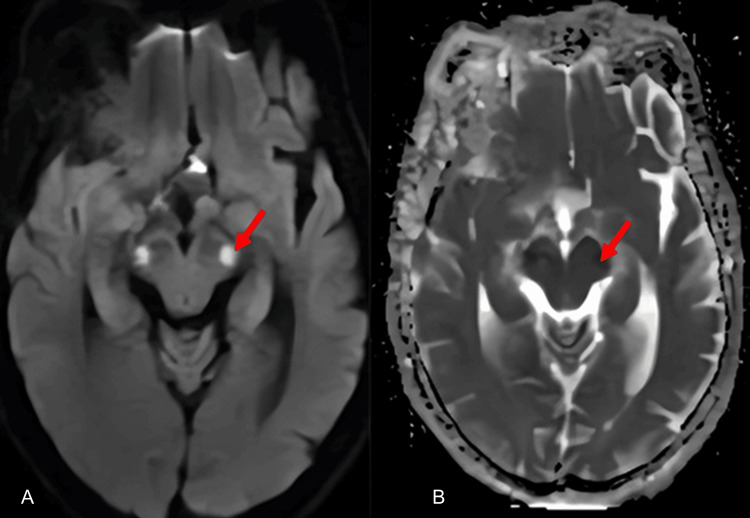
Neurological work-up findings Axial diffusion-weighted images (A) and apparent diffusion coefficient (ADC) (B) showing restricted bilateral pontine diffusion (red arrow). Diffusion weighted axial images (b=1000) and ADC showing a bilateral pontine restricted diffusion.

**Figure 2:**
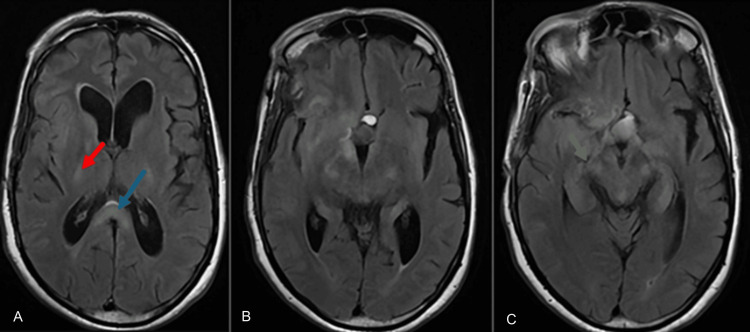
Axial T2-FLAIR MRI findings Axial T2-FLAIR MRI demonstrates hyperintensity along the pyramidal tracts. A: in the posterior limb of the internal capsule (red arrow) and in the splenium of the corpus callosum (blue arrow); B: showing the downward continuity of the signal abnormality from the internal capsule; C: extending further into the pontine region (green arrow). FLAIR: fluid attenuated inversion recovery

**Figure 3:**
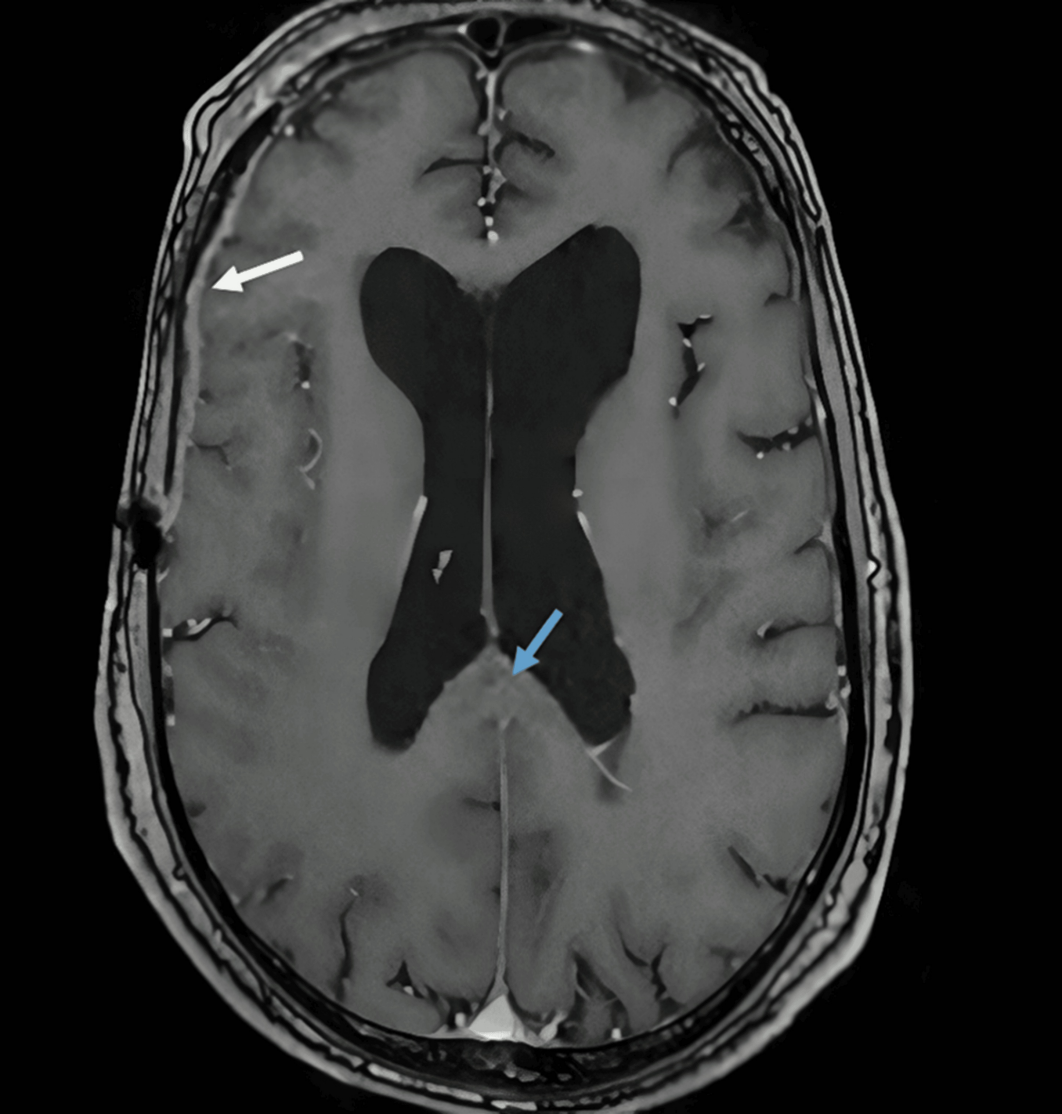
Neurological work-up findings Mild T1 signal hyperintensity not enhanced by contrast, seen in the pyramidal tracts, particularly in the splenium of the corpus callosum (blue arrow), with meningeal thickening (white arrow) around the right cerebral hemisphere with contrast enhancement, mainly in the frontotemporal region.

A CT scan of the brain without injection of contrast medium and a lumbar puncture were carried out as a matter of urgency, with no abnormalities. On the second day of hospitalization, the neurological work-up was completed by cerebral magnetic resonance imaging, which revealed centropontic and extrapontic myelinolysis of the pyramidal fascicles and cerebellar peduncles, and cortico-subcortical myelinolysis in the pre-central and post-central gyri, with thickening and contrast of the right cerebral hemisphere (Figures 1-3).

Given the clinical presentation, appearance, and topography of the lesions, the diagnosis of centropontine and extrapontine myelinolysis was established. A urinary ionogram was ordered, showing a low natriuresis of 38 mmol/24 h and a urinary osmolarity of 240 mOsmol/l. Water deficit was calculated at six liters. Initial management was based on rehydration with plain water and 0.9% saline via gastric tube, with reintroduction of desmopressin at a dose of 120 µg per day in two sublingual doses. The evolution was marked by a worsening respiratory condition with swallowing difficulties on the third day, necessitating intubation and mechanical ventilation with a weaning tracheotomy on the fourth day. On the ninth day, the neurological deficit improved, and natremia, natriuresis, and urinary osmolarity returned to 145 mEq/L (135-145 mEq/L), 80 mmol/d (50-300 mmol/l), and 290 mOsmol/l (280-305 mOsmol/l), respectively, with normalization of renal function (Table 1). The patient was discharged on day 14 on 90 µg desmopressin daily. Follow-up one month after discharge from the hospital showed good clinical evolution with recovery of motor deficit: 5/5 in both upper limbs and 4/5 in both lower limbs, disappearance of dysarthria and meningeal stiffness, and normalization of natremia to 138 mEq/L under 90 µg of desmopressin per day. A follow-up at six months after discharge showed complete recovery of deficits with a strictly normal neurological examination and natremia at 140 mEq/L under the same dose of desmopressin.

## Discussion

Osmotic demyelination syndrome is a rare condition involving the destruction of myelin sheaths, particularly in the pontine region, hence its former name of centropontine myelinolysis [7]. It results from symmetrical rupture of myelin sheaths in areas where white matter is exposed to osmotically active substances removed from edematous gray matter, namely the crossed cerebellopontine fibers within the pontine nuclei and other gray matter regions containing highly myelinated fibers, such as the cerebellum, lateral geniculate body, external and extreme capsules, corpus callosum, striatum, thalamus, and neocortical gray matter-white matter junctions [4,6,8]. The theory behind this is a decreased ability to adapt to strong variations in serum osmolality [9]. Subsequently, a hyperosmotic environment leads to glial dehydration and apoptosis, followed by demyelination [10]. There is a well-documented link between these disturbances and rapid osmolar variations, in particular precipitated sodium correction in patients with hyponatremia [2]. However, its appearance in the context of other osmotic conditions, including hyper- and hypoglycemia and hypernatremia, is increasingly recognized [6]. This syndrome may be favored by chronic alcohol intoxication, malnutrition, hepatopathy, or hypokalemia [11], leading to a special susceptibility of glial cells to osmotic variations.

Osmotic demyelination syndrome due to hypernatremia is rarely reported in the literature. To our knowledge, following the two cases reported by Börnke et al. in 2014 [12] and Gratieux et al. in 2020 [13], this is the third case of centropontine and extrapontine myelinolysis secondary to acute severe hypernatremia complicating the abrupt discontinuation of desmopressin prescribed as part of the treatment of central diabetes insipidus complicating neurosurgery.

The clinical picture may be nonspecific, depending largely on the area of the brain involved in demyelination. It may be associated with flaccid quadriparesis, damage to the cranial pairs (dysphagia, dysarthria, or diplopia, etc.), ataxia, extrapyramidal manifestations, tremors, hyperreflexia, convulsions, obnubilation, and coma [11]. In view of the rarity and limited specificity of clinical symptoms, cerebral magnetic resonance imaging, with its increased sensitivity in detecting demyelinating lesions, is playing a key role in the diagnosis of osmotic demyelination syndrome. Initial images may be normal, with obvious changes on repeated evaluation, which is why most experts suggest repeating the MRI about two weeks later if initial results are negative. The severity of radiological lesions often does not correspond to the severity of clinical signs or symptoms. Typical images include symmetrical, non-inflammatory, demyelinating lesions of the pontine and/or extrapontine structures [2] visualized as a hypersignal on T2-weighted sequences and as a hyposignal on T1-weighted sequences. The most frequent sites of extrapontine myelinolysis are the thalamus, basal ganglia, midbrain, subcortical white matter, and cerebellum [14]. In patients with hypernatremia, extrapontine lesions are more frequent than centropontine lesions [6].

Once diagnosed, this condition must be treated as a matter of urgency. Treatment is mainly symptomatic, aiming firstly to correct the underlying metabolic disorder, in our case, hypernatremia, and secondly to implement supportive actions to avoid the effects of brainstem dysfunction. For the treatment of hypernatremia, it is essential to be able to restore extracellular volume (ECV) by correcting circulating blood volume first and foremost [15,16] in the event of decreased ECV. After that, plasma hypertonicity is corrected by infusion of hypotonic fluid. The speed of this correction depends on the presence of clinical symptoms. If necessary, rapid correction should be made intravenously (0.45% saline, 5 or 2.5% glucose) or per os with water. The decrease in plasma tonicity should not exceed 1 mmol per liter per hour [16,17]. The use of the water deficit formula is recommended; however, the need for close hourly monitoring of natremia is a practice to be facilitated, and the patient must be able to be monitored in a specialized unit [18]. In our case, the hypernatremia was secondary to excessive urinary losses in the setting of maltreated central diabetes insipidus, and so the administration of desmopressin, in conjunction with correction of the fluid deficit, was necessary to correct the hypernatremia. In addition, the patient needed supportive care to avoid the effects of brainstem dysfunction, including inhalation pneumonia, urinary tract infections due to urinary retention, venous thrombosis, and amyotrophy resulting from prolonged immobilization.

On the other hand, the patient needed supportive measures to prevent the consequences of brainstem dysfunction, including inhalation pneumonia, urinary tract infections due to urinary retention, venous thrombosis, and amyotrophy resulting from prolonged immobilization.

Prognosis varies from complete recovery to persistent neuropsychiatric deficits and even death [19], and the severity of the disease at onset is not necessarily predictive of long-term outcome. A favorable outcome is reported in up to 50% of patients [20]. In a cohort of 83 Swedish patients with osmotic demyelination syndrome, the mortality rate at three months was 7%, with functional independence at three months of 60% [19].

## Conclusions

Osmotic demyelination syndrome remains the most serious complication associated with the rapid correction of dysnatremia. The clinical picture depends on the topography of the demyelinating lesions (centropontic/extrapontic), and MRI is the radiological examination of choice. Treatment is essentially symptomatic. Once often fatal, the prognosis of this condition has improved thanks to a better understanding of its pathophysiological mechanisms and main risk factors, as well as the availability of more effective diagnostic and therapeutic methods.
